# Residents’ Perceptions of Life in Assisted Living Facilities

**DOI:** 10.1093/geroni/igaf122.3196

**Published:** 2025-12-31

**Authors:** Katie Trainum, Heather Becker, Stephanie Morgan, Alexa Stuifbergen

**Affiliations:** University of Pennsylvania, Philadelphia, Pennsylvania, United States; The University of Texas at Austin, Austin, Texas, United States; The University of Texas at Austin, Austin, Texas, United States; The University of Texas at Austin, Austin, Texas, United States

## Abstract

Assisted living facilities (ALFs) account for half of all long-term care beds in the United States. Although focused on providing supportive and responsive personal care assistance, few studies have assessed residents’ perceptions of their ALFs, particularly as related to facility characteristics. In this study, 364 ALF residents living in 364 ALFs throughout a large southwestern state were interviewed about their perceptions of their ALFs using a closed-ended interview schedule. Their ALFs had an average of 42 beds (ranging from 5 to 177 beds) and occupancy rates from 10% to 100%. The average age of interviewed residents was 84.9 years (SD = 14.2), and most were non-Hispanic White women. Residents’ ratings of the availability of certain factors in their ALF were compared with ALF characteristics, such as occupied beds and on-site medical/nursing support. Most residents were satisfied with their ALF and reported they frequently had privacy, access to favorite foods, felt safe, and were respected by staff. Although most ratings had negligible correlations with facility characteristics, they were significantly correlated with concerns about staffing levels (r = -.23) and fear of retaliation (r = -.14). ALFs must address the delicate balance between resident acuity, safety, and autonomy. This balance becomes even more precarious as the medical complexity of ALF residents increases. Therefore, quality of life measures must take into account all these factors to provide a comprehensive understanding of residents’ perceptions of their ALF experience. Future studies should use well-developed measures to assess residents’ perceptions in diverse ALF settings.

